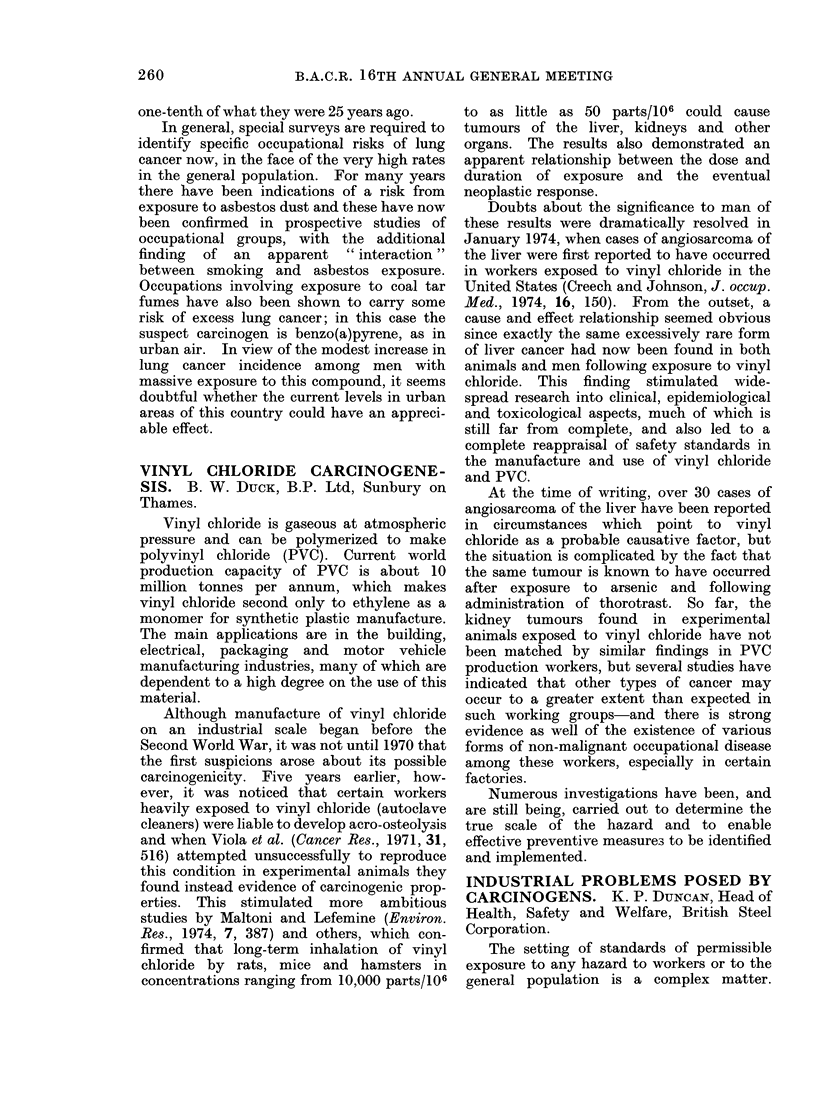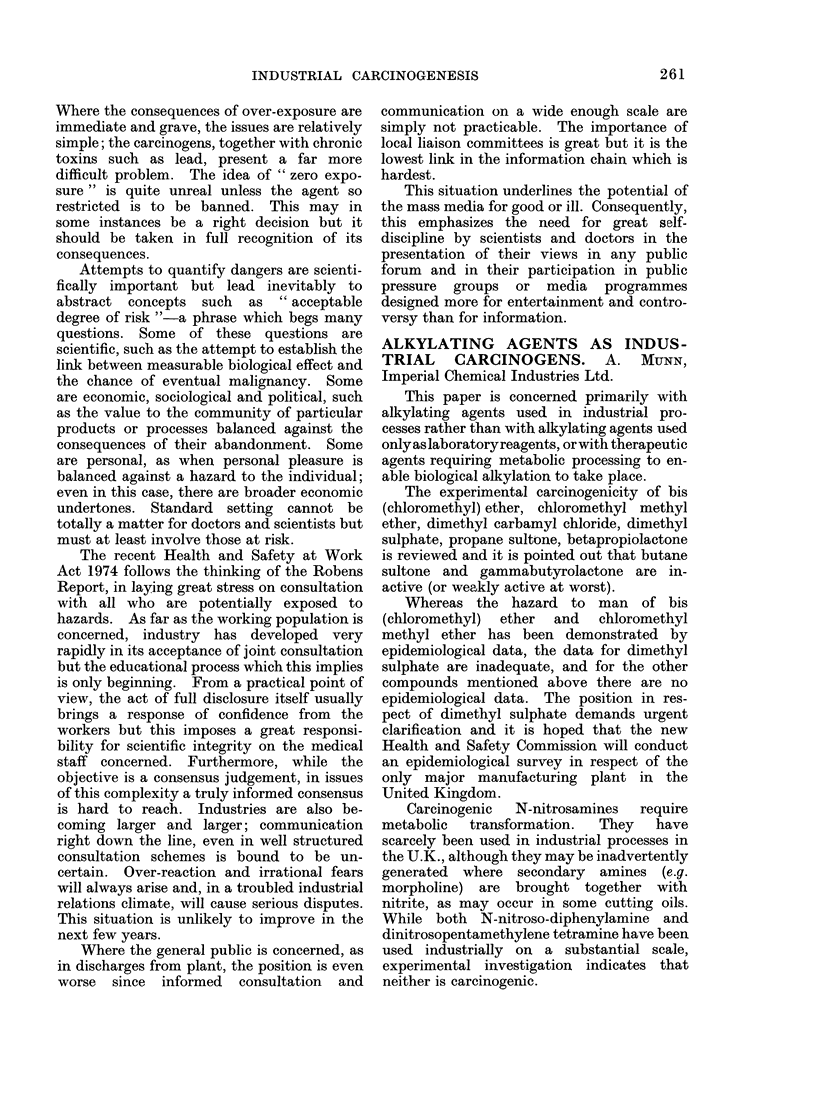# Proceedings: Industrial problems posed by carcinogens.

**DOI:** 10.1038/bjc.1975.209

**Published:** 1975-08

**Authors:** K. P. Duncan


**INDUSTRIAL PROBLEMS POSED BY CARCINOGENS.** K. P. Duncan, Head of Health, Safety and Welfare, British Steel Corporation.

The setting of standards of permissible exposure to any hazard to workers or to the general population is a complex matter.

Where the consequences of over-exposure are immediate and grave, the issues are relatively simple; the carcinogens, together with chronic toxins such as lead, present a far more difficult problem. The idea of " zero exposure " is quite unreal unless the agent so restricted is to be banned. This may in some instances be a right decision but it should be taken in full recognition of its consequences.

Attempts to quantify dangers are scientifically important but lead inevitably to abstract concepts such as " acceptable degree of risk "—a phrase which begs many questions. Some of these questions are scientific, such as the attempt to establish the link between measurable biological effect and the chance of eventual malignancy. Some are economic, sociological and political, such as the value to the community of particular products or processes balanced against the consequences of their abandonment. Some are personal, as when personal pleasure is balanced against a hazard to the individual; even in this case, there are broader economic undertones. Standard setting cannot be totally a matter for doctors and scientists but must at least involve those at risk.

The recent Health and Safety at Work Act 1974 follows the thinking of the Robens Report, in laying great stress on consultation with all who are potentially exposed to hazards. As far as the working population is concerned, industry has developed very rapidly in its acceptance of joint consultation but the educational process which this implies is only beginning. From a practical point of view, the act of full disclosure itself usually brings a response of confidence from the workers but this imposes a great responsibility for scientific integrity on the medical staff concerned. Furthermore, while the objective is a consensus judgement, in issues of this complexity a truly informed consensus is hard to reach. Industries are also becoming larger and larger; communication right down the line, even in well structured consultation schemes is bound to be uncertain. Over-reaction and irrational fears will always arise and, in a troubled industrial relations climate, will cause serious disputes. This situation is unlikely to improve in the next few years.

Where the general public is concerned, as in discharges from plant, the position is even worse since informed consultation and communication on a wide enough scale are simply not practicable. The importance of local liaison committees is great but it is the lowest link in the information chain which is hardest.

This situation underlines the potential of the mass media for good or ill. Consequently, this emphasizes the need for great self-discipline by scientists and doctors in the presentation of their views in any public forum and in their participation in public pressure groups or media programmes designed more for entertainment and controversy than for information.